# A portfolio analysis of autism research funding in Aotearoa New Zealand 2007–2021

**DOI:** 10.1177/13623613231155954

**Published:** 2023-02-19

**Authors:** Lisa Marie Emerson, Elizabeth Pellicano, Ruth Monk, Melissa Lim, Jessica Heaton, Laurie McLay

**Affiliations:** 1 University of Canterbury, New Zealand; 2 Macquarie University, Australia; 3 University College London, UK

**Keywords:** autism research, autistic community, funding, research priorities

## Abstract

Previously documented global trends in autism research funding have been skewed towards biology research, which is at odds with the priorities expressed by autistic and autism community members. We aimed to document the areas of autism research that have previously been funded in Aotearoa New Zealand, and to explore the views of the autistic and autism communities on this funding distribution. We searched for research grants awarded to autism research in Aotearoa New Zealand between 2007 and 2021. We categorised the funding for autism research to enable comparison to that previously documented in other countries. We elicited the views of the autistic and autism communities in relation to the funded autism research, through an online survey and a series of focus groups. The largest proportion of money and number of grants was awarded to biological research. Community members expressed dissatisfaction with this pattern of funding, and noted that it does not address the needs and priorities of the autistic community. Community members suggested that the funding pattern indicated a lack of autistic consultation and engagement in research design and funding allocation. The priorities of the autistic and autism communities need to be considered by researchers and funders alike. We discuss how autistic inclusion in research can be supported through decision-making regarding funding and ethics relating to autism research.

**Lay Abstract:**

We aimed to document the areas of autism research that have previously been funded in Aotearoa New Zealand. We searched for research grants awarded to autism research in Aotearoa New Zealand between 2007 and 2021. We compared the funding distribution in Aotearoa New Zealand to other countries. We asked people from the autistic community and broader autism community whether they were satisfied with this funding pattern, and whether it aligned with what is important to them and to autistic people. We found that the majority of funding for autism research was awarded to biology research (67%). Members of the autistic and autism communities were dissatisfied with the funding distribution, and expressed a lack of alignment with what is important to them. People from the community indicated that the funding distribution did not address the priorities of autistic people, and that it indicated a lack of engagement with autistic people. Autism research funding needs to reflect the priorities of the autistic and autism communities. Autistic people need to be included in autism research and related funding decisions.

Previous research has reported on the allocation of autism research funding in the United Kingdom (UK; L. Pellicano et al., 2013), the United States (US; Harris et al., 2021; Office of Autism Research Coordination [OARC] & National Institute of Mental Health, 2017, 2019; Singh et al., 2009), Canada (Krahn & Fenton, 2012) and Australia (den Houting & Pellicano, 2019). Generally, the findings were consistent across countries, with the largest proportion of autism research funding (27%–65%) being allocated to biological research (e.g. biological differences associated with autism). In 2019, the Interagency Autism Coordinating Committee (IACC et al., 2019) compared autism research funding awarded across the US, UK, Canada and Australia in 2016. The combined data from these four countries indicated a skew towards basic science with 36% of funded projects categorised as biological research, and 23% as research on causes and risk factors (e.g. genetics, epigenetics and the environment). This skew was also reflected in relation to the amount of funding awarded.

In the US, the IACC (2017, 2020) makes budget recommendations to the government in relation to autism research. Following the identified bias towards biology and basic science, the IACC highlighted priority areas that required additional funding, including interventions, evidence-based services and lifespan issues. In a subsequent funding portfolio analysis, Harris et al. (2021) reported that between 2017 and 2019, funding for autism research on biology and risk factors declined, but that a disproportionate amount of funding was still allocated towards these two areas (55% 2017; 43% 2018; 39% 2019). There was comparably less funding allocated to the three priority areas highlighted by the IACC (20%, 5% and 2.5% allocated towards research into interventions and treatments, services and lifespan issues, respectively). Similarly, between 2008 and 2018, only 9% of autism research funded by the National Institutes of Health (NIH) was categorised as services research (Cervantes et al., 2021). Thus, within the US, there is a persistent misalignment between the strategic plan and budget recommendations set out by the IACC and the actual funding allocation for autism research.

In recent years, there have been increasing calls for investment in autism research that directly benefits the community, with reports that have documented community driven priorities for autism research. In the US, Frazier et al. (2018) reported the findings of an online survey of ‘autism stakeholders’ (including autistic people, family, researchers and practitioners); this wider autism community prioritised applied research relating to co-occurring conditions, health and wellbeing, adult transition and lifespan issues. E. Pellicano and colleagues (2014) reported the perspectives of the autistic and autism communities from a mixed methods study (interviews, focus groups, online survey). These community priorities highlighted a need for autism research with practical implications for services and supports for autistic people, including everyday skills, employment and post-diagnostic support, as well as knowledge about autism (e.g. practitioner training and public awareness). In a systematic review of the research priorities of the autism community, Roche et al. (2021) highlighted a common international priority for research that results in real-world change for autistic people (e.g. skills development training from childhood into adulthood and employment; physical health, wellbeing and mental health), with an emphasis on research across the lifespan (e.g. expertise, coordination, availability and accessibility of services across the lifespan). The discrepancy between the funding pattern for autism research (i.e. the bias towards biological research) and what the autistic and autism communities themselves prioritise for research has also been highlighted. However, only one of these studies specifically asked the autistic and autism communities about their views of the funding portfolio in their country. E. Pellicano et al. (2014) found that all community groups, including researchers, predominantly reported dissatisfaction with the lack of breadth and skewed nature of the UK funding portfolio for autism research, indicative of a misalignment between what is being researched and their own priorities.

Different countries will have their own unique demographic and sociocultural context, as well as differing models of funding and service delivery. In Aotearoa New Zealand (NZ), autism research is predominantly funded by government through the Health Research Council, Ministry for Business, Innovation and Enterprise and Ministry of Education, or through independent and donor organisations, including the Royal Society and regional Medical Research Foundations. There is currently no coordinating or oversight body that makes budget recommendations in relation to autism research in Aotearoa NZ. It is unclear what autism research has previously been funded in Aotearoa, and how this is perceived by the autistic and autism communities. We aimed to conduct the first comprehensive appraisal of funded autism research in Aotearoa NZ. We used the IACC Strategic Plan research questions to classify the types of research projects funded in between 2007 and 2021, and compared this categorisation of Aotearoa NZ autism research with those previously documented for the US, UK, Canada and Australia (including comparison of funding amount with adjustment for gross domestic product (GDP)). Building on previous research, we also aimed to understand the perspectives of the autistic and autism communities in relation to the funded research, including their satisfaction and perceived alignment with their own priorities for autism research (online survey) and broader views (focus groups).

## Method

### Search strategy for research grants

Following previous studies (Daniels & Warner, 2018; den Houting & Pellicano, 2019), we searched for autism relevant research grants using the Dimensions Plus database (https://app.dimensions.ai), utilising search terms: Autism, Autism Spectrum Disorder, Autis*, Autistic, ASD, Asperger, Asperger’s, AUTS1, AUTS2, ASC, PDD-NOS, PDD NOS and PDDNOS. The Dimensions Plus database currently lists awards from four key Aotearoa NZ funders: Health Research Council of New Zealand (HRC), Royal Society of New Zealand (RSNZ), Ministry of Business Innovation and Employment (MBIE) and Auckland Medical Research Foundation (AMRF). We also searched annual reports, and previous grants listed on webpages for other relevant funders, including regional Medical Research Foundations and philanthropic organisations (e.g. Curekids; New Zealand Council for Education Research). Where required, we contacted funders to request this information if it was not publicly available. A small number of non-government funders were not prepared to disclose this information or did not maintain records of their allocation of grant funding.

**Figure 1.**
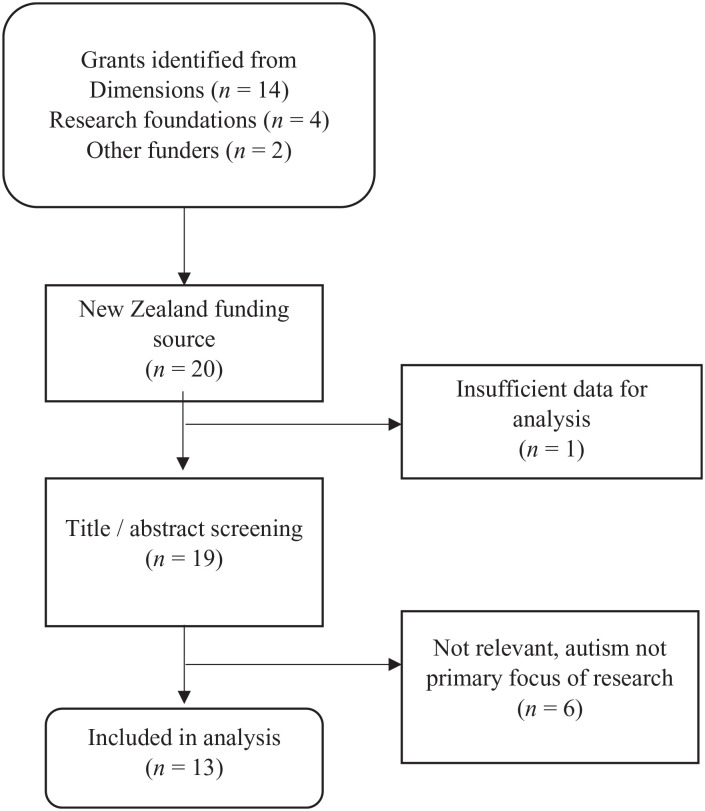
Flow diagram of search process.

Searches were limited to research grants awarded by funders in Aotearoa NZ since 2007 (date of first entry on Dimensions Plus) and up to 2021 (searches conducted May 2021). Research grants that were (1) primarily focused on autism and (2) awarded by a funder based in Aotearoa NZ were eligible for inclusion. Two independent researchers reviewed the title and abstract/summary of the research grants; those that were not obviously relevant were flagged for further review and consensus agreement by one or two co-authors (L.M.E. and L.M.). Figure 1 documents this search process. We identified a total of 13 research grants for inclusion.

#### Coding and analyses

The identified research grants were coded for research topic according to the seven research priorities set out in the IACC strategic plan (IACC, 2017; see den Houting & Pellicano, 2019; E. Pellicano et al., 2014): (1) *Diagnosis and Screening*, (2) *Biology*, (3) *Causes and Risk Factors*, (4) *Treatments and Interventions*, (5) *Services*, (6) *Lifespan Issues* and (7) *Infrastructure and Surveillance*. The language utilised is taken from the IACC (2017) strategic plan. Each grant was blind coded by two raters. Initial inter-rater agreement was 92%. A third independent rater reviewed the first and second raters’ codes to resolve disagreements, with the final code reported here.

Utilising the coding of research topics described above, we documented the amount of funding, the number of projects and funding by year. Cash investments only were included in our analyses; in-kind contributions to the research were not included. We compared the categorised funding in Aotearoa NZ to those previously documented from the UK, Canada, the US and Australia, using a snapshot of funding from 2016 as per the data reported by the OARC and National Institute of Mental Health (2019). The year 2016 was selected as the OARC report focused on funding awarded in these four countries in 2016 alone. Funding amount was adjusted to account for differences in GDP between countries, and is reported as a percentage of GDP. In addition, the unique cultural context of Aotearoa NZ was considered: grants were screened to determine the proportion of funding allocated to Māori research.

### Eliciting community views

We obtained the perspectives of the autistic and autism communities in Aotearoa NZ on the categorised funding in Aotearoa NZ through an online survey and a series of focus groups. All participating people provided informed consent prior to taking part in either the online survey (via study webpage) or the focus groups (via electronic consent form). Descriptive data pertaining to the categorised funding were presented to participants in a 5-min PowerPoint presentation (pre-recorded video and transcript in online survey), including pie charts to show the distribution of funding between categories.

#### Online survey

An online survey was distributed via community networks, including autism organisations in Aotearoa NZ, practitioner and research networks and social media. The survey was open to all adults in the community who identified an interest in autism research, including autistic people and people in the broader autism community (e.g. family and practitioners) residing in Aotearoa NZ. The survey was hosted on Qualtrics between November and December 2021. After viewing the video-recorded presentation of funded autism research in Aotearoa NZ, survey respondents rated two questions on a 5-point Likert-type scale to indicate *(1) their satisfaction with the documented funding (1, very dissatisfied – 5, very satisfied)* and *(2) the extent to which the funded research aligned their own priorities for autism research (1, not at all – 5, almost exactly).* For each question, participants were able to select ‘prefer not to answer/don’t know’.

Data from complete survey responses were included (early closure of the survey was deemed withdrawal from the study); a total of 293 incomplete responses were removed. A total of 450 people completed the survey (see Table 1): 151 (34%) survey participants identified their primary interest in autism research as an autistic person; 194 (43%) as a parent or carer of an autistic person; 9 (2%) as a family member of an autistic person (1 child of an autistic person; 3 siblings; 2 spouse/partners and 5 extended family); 52 (11.5%) as a healthcare/disability practitioner (e.g. speech and language therapist, psychologist and support worker); 45 (10%) as an educator (e.g. teachers and lecturers) and 11 (2.4%) as an autism researcher. Of the 151 people who identified their primary interest in autism research as an autistic person, a minority reported co-occurring learning, intellectual or developmental disability (*n* = 28; 18.5%), speech and language disorder (*n* = 24; 15.8%), coordination or motor difficulty (e.g. dyspraxia) (*n* = 53; 35%), specific learning disability (e.g. dyslexia, dyscalculia and dysgraphia) (*n* = 30; 19.9%) or attention deficit/hyperactivity disorder (*n* = 42; 27.8%). Across all groups, 175 (39%) people identified as autistic (primary or secondary interest in autism research). Family members of an autistic person were grouped with the parent/carer group to create a whānau (family) group for the purposes of this study (similar to E. Pellicano et al., 2014).

**Table 1.** Demographic information of participants (online survey and focus groups).

	Online survey					Focus groups				
	Autistic adults	Whānau	Researchers	Healthcare/disability practitioners	Education practitioners	Autistic adults	Whānau	Researchers	Healthcare/disability practitioners	Education practitioners
Gender										
Female/woman	73	171	7	44	43	6	9	5	10	6
Male/man	38	26	3	5	1	4	3	2	0	1
Gender diverse	29	1	0	2	0	3	0	0	0	0
Age (years)										
18–21	24	1	0	0	0	1	0	0	0	0
22–30	33	9	0	10	1	2	2	2	1	0
31–40	39	45	5	10	5	5	2	2	1	2
41–50	25	80	2	10	13	2	4	2	3	2
51–60	21	51	3	13	19	1	2	1	4	1
61+	9	16	1	9	7	2	1	0	1	2

A total of 15 survey participants did not disclose their gender. One focus group family member did not disclose their age. Information on age was not recorded for individuals who participated in focus groups for Māori and Pacific peoples.

Survey data were analysed by primary interest group using descriptive statistics, including percentage endorsement rates, mean values and standard deviations (SDs), and mode response for satisfaction and alignment questions. Analyses of variance (ANOVAs) and Tukey’s Honestly Significant Difference (HSD) assessed between-group differences.

#### Focus groups

We conducted 11 focus groups with people recruited from the community. Individuals responded to a study advertisement distributed through the project team and advisory group networks and posted on social media (e.g. Facebook groups). Thus, a convenience sample of individuals from the autistic and autism communities took part in the focus groups. Recruitment for the focus groups was independent of recruitment for the online survey. Individuals were recruited to focus groups based on their interest in autism research; these groups were exclusive to those with the same interest in autism research (e.g. autistic adults only): autistic adults (*n* = 13); parents, family and whānau of an autistic person (*n* = 12); autism researchers (*n* = 7); healthcare/disability practitioners (*n* = 10) and education practitioners (*n* = 7) (see Table 1 for demographic information). Two exclusive focus groups were also conducted for whānau Māori (*n* = 3 mothers of autistic children) and Pacific peoples (*n* = 3; two fathers and one mother of autistic children). Individuals with multiple interests in autism research (e.g. an autistic researcher and an autistic parent of an autistic person) chose which group they would prefer to be in. Focus groups were 60 min, and conducted online via Zoom. Participants could contribute verbally and/or via the chat function; all contributions were included in the analyses. Groups were facilitated by a member of the research team. The autistic adults’ focus groups were facilitated by an autistic researcher. A second researcher took notes and provided a summary of the key discussion points at the end of the focus groups.

Following the 5-min presentation of the categorised funding, the facilitator asked two questions to prompt discussion: *(1) Do you think autism research funding in Aotearoa New Zealand currently reflects your priorities? (2) To what extent do you feel autism research funding is representative of the priorities of the Autistic community?* (see E. Pellicano et al., 2014, for a similar methodology).

Focus groups were transcribed verbatim and followed Braun and Clarke’s (2006, 2019) method for reflexive thematic analysis. We used an inductive (bottom-up) approach (i.e. without integrating the themes within any pre-existing coding schemes or preconceptions of the researchers) to identify patterned meanings within the data set specifically related to research priorities. The broader team brought a diverse range of perspectives to bear on the analysis, from disciplines of psychology, education and health, as well as positionalities as an autistic researcher (R.M.). Analysis, primarily conducted by a core team of autistic (R.M.) and non-autistic researchers (M.L. and L.M.E.), included prolonged and intense engagement with the data and a collaborative and deeply reflexive process, recognising that themes are an active construction of what we as researchers aimed to know, our assumptions and background and the nature of the data themselves.

To begin, the analytic process started with data familiarisation, which involved ML reading and re-reading the focus group transcripts, debriefing and discussing potential codes with R.M. and L.M.E. before applying the codes to the entire data set. In discussion, the analysis team identified potential themes, focusing on semantic features of the data (staying close to participants’ language) and resolving discrepancies. The final themes were determined through a process of with the broader team. Analysis was therefore iterative and reflexive, moving backward and forward between data and analysis, considering researchers’ potential biases and incorporating insights from the analytic process.

### Community involvement

The project team (authors) included autistic and non-autistic researchers. The design of the online survey and focus groups were informed by consultation with and feedback from two advisory groups. The Autistic Advisory Group (AAG) included autistic adults with a range of secondary interests in autism (e.g. parent, researcher and advocates). The Partnership Advisory Group (PAG) included representatives from the AAG, as well as parents, practitioners, autism organisations and researchers. The advisory groups included Māori members and Pacific peoples. These groups met with the project team on three occasions to advise on the design of the community consultation activities, including format of focus groups, design of survey questions, recruitment and dissemination, analysis and reporting of data.

Ethical approval for this study was granted by the Human Research Ethics Committee, at the University of Canterbury (Ref. 2020/134). All participants provided informed consent prior to taking part.

## Results

### Research grants

Table 2 reports the number of grants and total funding by the IACC strategic plan priority areas. Between 2007 and 2021, 13 autism research grants were awarded in Aotearoa NZ. Total investment across these grants was NZD$3,753,742. The majority of grants were awarded by regional medical research councils (*n* = 5), the HRC (*n* = 4) and the RSNZ (*n* = 3). The largest proportion of funding was awarded by the RSNZ (NZD$1,809,000), followed by the HRC (NZD$1,640,000).

**Table 2.** Number of grants and total funding of autism specific grants in Aotearoa New Zealand 2007–2021.

IACC research priority area	Number of grants (%)	Total funding NZD$ (%)
Biology	8	2,496,650 (67%)
Treatments and interventions	3	1,181,000 (32%)
Diagnosis and screening	1	47,092 (1%)
Infrastructure and surveillance	1	29,000 (<1%)

IACC: Interagency Autism Coordinating Committee.

The largest number of grants and largest proportion of funding of total funding was allocated to *Biology* research, followed by research on *Treatments and Interventions*. No funding was allocated to research relating to *Causes and Risks*, *Services* or *Lifespan Issues*. The largest singular grant was NZD$1,200,000 (*Biology*); the smallest singular grant was NZD$4000 (*Biology*). The median grant size was NZD$159,000.

Over the 14-year period, there were seven non-consecutive years of no autism research grants. The maximum number of grants awarded in any given year was three (in both 2013 and 2016). The largest investment was made in 2017 with NZD$1,361,000 funding between two research grants (NZD$1,200,000 *Biology*; NZD$161,000 *Interventions and Treatments*). No grants related to Māori research were identified.

#### International comparison

Three grants were awarded to autism research in Aotearoa NZ in 2016, totalling NZD$506,092. Table 3 provides a direct comparison (all USD$) with data extracted from the 2016 International Autism Spectrum Disorder Research Portfolio Analysis report (OARC & National Institute of Mental Health, 2019) pertaining to the funding portfolio of the UK, Canada, the US and Australia. As a percentage of GDP, grant funding awarded in Aotearoa NZ was the lowest. Two of the three grants awarded to autism research in Aotearoa NZ in 2016 were categorised as *Biology* research (third was *Diagnosis and Screening)*, consistent with the dominance of biology research in other countries (64% UK, 35% US, 40% Canada).

**Table 3.** Comparison of total funding and as a percentage of GDP for Aotearoa New Zealand, the UK, the United States, Canada and Australia in 2016.

Country	Total funding 2016 USD$ (number of grants)	% GDP, scientific notation
Aotearoa New Zealand	506,092 (3)	2.68912e–4
The UK	14,848,929 (59)	5.51185e−4
The US	364,435,254 (1360)	1.947810e−3
Canada	10,719,396 (74)	7.01531e−4
Australia	5,854,451 (59)	4.84239e−4

GDP: gross domestic product; UK: United Kingdom; US: United States.

### Eliciting community views

#### Online survey

**Figure 2.**
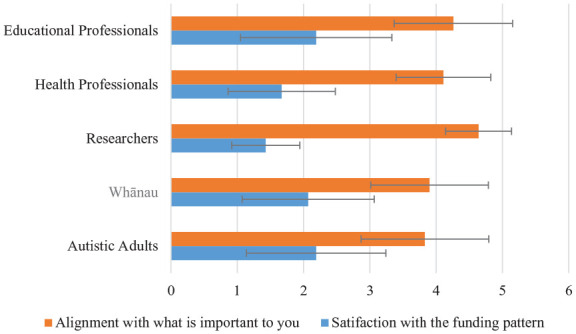
Mean ratings for funding portfolio questions according to primary interest group (error bars indicate standard errors).

Figure 2 shows the percentage endorsement of satisfaction and alignment ratings by respondent group; mean ratings (and SDs) are reported in Table 4.

**Table 4.** Participants’ mean ratings for funding portfolio questions according to primary interest group.

	Autistic adults		Whānau		Researchers		Health professionals		Educational professionals		Group effect			Tukey’s HSD^a^
	Mode	M (SD)	Mode	M (SD)	Mode	M (SD)	Mode	M (SD)	Mode	M (SD)	*F* (df)	*p*	Effect size *n*^2^	
Satisfaction with the funding pattern	1	2.19 (1.05)	1	2.07 (0.99)	1	1.43 (0.51)	2	1.67 (0.81)	2	2.19 (1.14)	3.70 (4, 410)	0.006	0.035	Autistic adults > healthcare/disability practitioners (*p* = 0.03)
Alignment with what is important to you	1	3.83 (0.96)	2	3.90 (0.89)	2	4.64 (0.50)	3	4.11 (0.71)	2/3^b^	4.26 (0.89)	4.19 (4, 397)	0.002	0.040	Researchers > autistic adults (*p* = 0.01) Researchers > whānau (*p* = 0.02)

HSD: honest significance difference.

Post hoc tests only reported if *p* < 0.05.

a Tukey’s HSD based on comparison of mean values and SDs.

b Multiple modes.

The mode response for satisfaction suggested that all groups were dissatisfied with the categorised funding. There were significant differences between-group in satisfaction ratings (*F* = 3.697 (4, 410), *p* = 0.006, η^2^ = 0.035). Post hoc Tukey’s HSD tests confirmed that the healthcare/disability practitioner group satisfaction ratings were significantly lower (less satisfied) than the autistic adult group (*p* = 0.03, 95% confidence interval (CI) = 0.03–0.99).

The mode response for alignment with what is important to you indicated misalignment for most groups. There were significant between-group differences in alignment ratings (*F* = 4.19 (4, 397), *p* = 0.002, η^2^ = 0.04). Post hoc Tukey’s HSD tests confirmed that the researcher group ratings were significantly higher (greater alignment) than the autistic adult (*p* = 0.01, 95% CI = 0.13–1.49) and the whānau (*p* = 0.02, 95% CI = 0.07–1.42) groups’ ratings.

#### Focus groups

Collectively, all groups expressed disappointment and disagreement with the funding for autism research in Aotearoa NZ, with the exception of one researcher. The community reaction was neatly summarised by one of the autistic adults who stated that they would like ‘other researchers and funders to hear that, no, we are not happy’ with the current funding distribution.

##### Non-autistic priorities

All groups agreed that the funded autism research was not representative of the priorities of the autistic community. Specifically, the distribution of funding between categories of research was considered to be unbalanced. In reference to biology research, one autistic adult commented that this kind of research did not align with their future aspirations: ‘It’s not our future, it’s not young autistics’ future, and we don’t want to stay in that, we don’t want them to stay in that’. The researcher group considered possible reasons why the funding was skewed towards biology research. One researcher commented on the expense of such research, ‘Like the proportion of money that goes into some of these categories are, they seem unbalanced, but . . . genetics research is expensive’. Another researcher highlighted the pressure to tailor research towards what has an increased chance of receiving funding: ‘It is kind of one of those things where when you’re applying for funding, you know that you have to play to what you think that will get funded, rather than the research that you want to do’. Researchers also highlighted the need for research to be meaningful and giving back to the autistic community. One researcher questioned if current autism research is ‘benefiting the scientific world, who understands better, but does not have any impact on the life of autistic people’. Across all groups, it was noted that the funding was a poor reflection of the real needs of the autistic community.

All focus groups reflected that the funding seemed to represent a deficit view of autism: ‘[it] has a feeling of trying to fix something rather than embracing and facilitating diversity’ (Education practitioner). All focus groups discussed the need for autism acceptance and for research funding to focus on ‘making autistic lives better’ (Parent/Carer), including the lives of parents and family. Across the discussions, there was resounding agreement that more funding should be directed towards research on *Screening and Diagnosis*, *Services* and *Across the Lifespan* categories.

##### Missing research

Several key areas of missing research were highlighted by participants, including research focused on the strengths of autistic people and ethnic and cultural understanding. There was reference to the ‘superpowers’ that autistics have, and wanting to see research that explores and celebrates the strengths and achievements of autistic people: ‘I think just seeing the incredible stuff that autistics do and the areas they go into when they are supported’ (autistic adult), ‘how people really thrive who have autism, and specifically with strengths in areas as well’ (Parent/Carer) and ‘wouldn’t it be amazing to see research examples of, like, autistic people’s resilience and ability to thrive’ (autistic adult).

All groups pointed out the importance of considering the ethnic and cultural context within Aotearoa NZ and highlighted that this was not reflected in the current funding for autism research. One autistic adult noted that there needed to be a greater ‘emphasis on autistic people of ethnic minority, including Tangata Whenua [original inhabitants of Aotearoa NZ]’. A Māori parent/carer spoke about the need for autism research to explore ‘having autism and being Māori. What does that mean for Māori?’, while a Pacific parent/carer highlighted the need for research to be ‘looking at strengths of autism and Pasifika culture’. A healthcare/disability practitioner noted that they work alongside a ‘large number of Asian families’ within the autistic community and highlighted the importance of considering immigrant populations. One autistic adult also commented on the significance of intersectionality in Aotearoa NZ, noting that ‘Autism doesn’t occur in isolation, what about social studies and experience-based research investigating rich combinations of autism and ADHD, autism and gender and sexual diversity, culture and ethnicity?’.

##### Lack of autistic involvement

Across the focus groups, participants highlighted the apparent lack of autistic involvement in research. One autistic adult commented, ‘it’s funding people who are not autistic and none of that money is going to autistic people to do their own research’. Another autistic adult echoed this sentiment and alluded to a systemic issue: ‘That’s the biggest problem that there’s a large amount of money going around . . . but none of it’s gotten into the pockets [for] autistic people to do the research themselves. It’s basically an oversight system’.

Other groups also noted that the categorised funding seemed to indicate a lack of engagement with the autistic community, and a lack of consideration of their needs and preferences. As one educator stated, ‘the funding profile indicated quite clearly how little autistic people are involved in the research, because I don’t think that would be the perspective that they are coming from at all’.

## Discussion

We documented previous funding for autism research in Aotearoa NZ over a 14-year period (2007–2021). We utilised the IACC strategic plan to categorise grants, and compared funding in Aotearoa NZ to the US, the UK, Canada and Australia. Between 2007 and 2021, 13 autism research grants were awarded in Aotearoa NZ, equating to a total investment of NZD$3,753,742. As a percentage of GDP, the amount of funding awarded was substantially less than the UK, Canada, the US and Australia in the 2016 comparison year. This GDP adjustment shows that difference in scale between the countries does not fully account for the lower grant funding documented in Aotearoa NZ. Nevertheless, consistent with international research, both the number of grants and total amount of funding for autism research were disproportionately allocated to *Biology* research. In this case, eight grants and a total of NZD$2,496,650 were awarded to *Biology* research, which constituted 67% of the total funding. Consistent with international trends, relatively few grants were awarded for *Treatments and Interventions, Diagnosis and Screening* and *Infrastructure and Surveillance*, and none focused on *Causes and Risks, Services* or *Lifespan Issues.*

Strikingly, the categorised funding included no grants relating to Kaupapa Māori (Māori research). Māori children are more likely to be diagnosed with autism than non-Māori (Ministry of Health [MOH], 2021; Tupou et al., 2021), and yet Māori are underserved by current models of healthcare delivery and face inequities in access to services and supports as well as healthcare outcomes (Kingi et al., 2017). To honour the commitment to Te Tiriti o Waitangi (Treaty of Waitangi), the specific needs of Māori must be prioritised. While funding bodies have set out to prioritise research that enhances Mātauranga Māori (Māori knowledge) and enhances Māori health outcomes, this commitment is not reflected in the current funding for autism research in Aotearoa NZ.

We sought the reactions to this categorised funding from members of the autistic and autism communities through an online survey and a series of focus groups. People broadly indicated dissatisfaction with the current funding and its misalignment with community preferences. Autistic adults and broader autism community members suggested that further resourcing should be allocated towards research into *Screening and Diagnosis, Lifespan Issues* and *Services.* Community members also emphasised the need for research that reflects the unique cultural and ethnic context of Aotearoa NZ. Participants commented that funding was not representative of the needs and priorities of the autistic community; that it instead highlighted a lack of autistic consultation and engagement in research design and funding allocation, and perpetuated a pathologising view of autism. These views reflect those evidenced in international research (E. Pellicano et al., 2014).

In the US and UK, research into the priorities of the autistic and autism communities for autism research has highlighted the need for a more applied and translational focus, including health and wellbeing, transition into adulthood, lifespan issues, employment and post-diagnostic support and knowledge of autism within society (Frazier et al., 2018; E. Pellicano et al., 2014). These findings were similarly reflected in a systematic review of community research priorities (Roche et al., 2021). Although the research priorities of autistic people and the autism community in Aotearoa NZ are not currently known (although research is underway; Emerson et al., under review), the current community reaction to the categorised funding in this study, and global perspectives on autism research priorities (Frazier et al., 2018; E. Pellicano et al., 2014; Roche et al., 2021), suggest that the funding allocation in Aotearoa NZ is not reflective of community needs and preferences.

Interestingly, community responses were generally consistent across all groups in the focus groups and survey, indicating dissatisfaction with the funding and perceived misalignment with their priorities. Education and healthcare/disability practitioners’ survey responses indicated that the research funding distribution was *not really* to *somewhat* representative of what is important to them (mode response). While this does not necessarily indicate endorsement of the funding distribution, it does suggest a slightly more favourable view when compared to the responses of autistic adults, whānau and researchers (mode responses: *Dissatisfied* and *Very Dissatisfied*). The perspectives of practitioners may be indicative of the context-specific needs identified in their role as service providers or in the case of medical practitioners, may reflect a more medically oriented paradigm adopted within their profession, and the orientation towards biological and medical aetiology and treatments.

There is a pressing need for services and supports that can meet the needs of autistic people across the lifespan and across settings (e.g. health, education and workplace). It is critical that these services are both effective and endorsed by autistic people. The lack of funding investment in this area of research will hinder the advancement of impactful translational research that is appropriate for the context of Aotearoa NZ. Currently, in Aotearoa NZ, the views of autistic people are not intentionally or routinely included in funding decisions. This is in spite of significant global progress in this area. Internationally, advisory committees/boards such as the IACC have been established to advocate for consistent levels, and equitable and appropriate distribution of autism research funding. These bodies have been tasked with developing and monitoring a strategic plan for autism research, evaluating and reporting on funding trends, elucidating research findings and directing future funding priorities. Likewise, research cooperatives such as the Autism Cooperative Research Centre in Australia promote inclusive, participatory research that is reflective of the priorities of autistic people. For the Autism CRC in particular, this has resulted in a relatively large proportion of funding being allocated towards *Infrastructure and Surveillance, Lifespan Issues* and *Treatments and Interventions*; a distribution that is more representative of the priorities of the autistic and autism communities (den Houting & Pellicano, 2019). In order to ensure that funding allocation in Aotearoa NZ is of direct benefit to the autistic community, it is timely for similar models to be adopted at a national level.

The findings of den Houting and Pellicano (2019) suggest that a top-down approach (i.e. leadership from funding organisations) combined with a bottom-up approach (i.e. partnership with the autistic and autism communities) is needed for funding to more adequately meets the needs of the autistic community (see also L. Pellicano et al., 2013). There is a growing movement towards including autistic people and their allies as partners in the research process (see Fletcher-Watson et al., 2019; Nicolaidis et al., 2011; E. Pellicano et al., 2011). Some (e.g. Chown et al., 2017) have outlined the requirements for inclusive autism research, which includes that autistic researchers define and validate the topics for investigation. In this way, the intention is that autism research is directly informed by the community that it seeks to benefit. Participatory research design of this kind refers to research methods that are inclusive to community members (Cargo & Mercer, 2008). In a recent examination of the views of autism research stakeholders on participatory research design, den Houting et al. (2021) reported a high level of enthusiasm for community engagement in autism research. However, actual autistic engagement was found to be inconsistent across the stages of the research process, with distinctly lower levels of engagement at the early (e.g. grant proposal writing and background research) and late (e.g. data analysis and dissemination) phases of research, compared to a concentration of community engagement during the stages of recruitment and amendments to data collection methods (e.g. interview and survey questions). Even more disappointing, less than half of researchers in this study reported that adjustments were made to the project in response to community engagement. This suggests that while autistic individuals are consulted at specific stages of the research process, their views are not integrated, and the power for decision-making still lies with researchers. While some of this may be down to the choice of individual researchers, there is also a large role-played by existing structures that support research.

Authentic and equitable engagement requires far more than just including or consulting with autistic people in individual research projects, but must extend to the decision-making across all phases of the research cycle. In other words, while the intentions and efforts of individual researchers are necessary, they are insufficient and can only extend so far. Indeed, in a recent review of autism science, E. Pellicano and den Houting (2022) outlined that ‘Commitment to autistic involvement cannot be left entirely up to individual researchers’ (p. 8), and that organisations and funding bodies have a duty to ensure autistic involvement within their structures and strategy.

The Health Research Council, Ministry of Business Innovation and Enterprise and the Ministry of Health (2019) in Aotearoa NZ, published the New Zealand Health Research Prioritisation Framework to guide health research investment decisions through to 2027. The core aims stipulated in this framework include partnering with communities to co-design supports and services that enhance physical and mental health and wellbeing, providing the necessary knowledge to improve health and disability services and optimisation of resources and ensuring that services reflect the needs of specific communities by understanding diverse perspectives, the sociocultural and historical underpinnings of these perspectives and the inequities experienced by marginalised groups. In the context of this study, it seems pertinent that funders take action to ensure that funding decisions are informed by, and reflect the views of autistic people specifically, as well as those who support autistic people.

Autistic involvement is needed within decision-making regarding funding and ethics relating to autism research. Such involvement could mean establishing advisory committees or cooperatives that centralise autistic expertise with the remit to contribute to scientific reviews and inform the decision-making processes around funding autism research. In order for autistic people to be authentically engaged in the research process, it is necessary that these environments are inclusive and accessible to autistic people. At a minimum, this will mean consideration of training and mentorship to autistic people so that they can successfully engage in each area of the research process. In addition, there is a need for parallel education for stakeholders in autism research (e.g. funders and ethics committees) to be cognisant of the principles and conditions of authentic community engagement in research. Funders of autism research also have a key role to play in how they encourage and expect community involvement during the granting process. In the UK, major funding bodies have published guidelines on the expectations of community involvement. For example, the UK Standards for Public Involvement (UK Public Involvement Standards Development Partnership, 2019) are promoted by the National Institute for Health Research (NIHR). Applications to grant funding schemes from the NIHR require researchers to detail how community members have been involved in the design of the proposed research, and how they will continue to be involved in the research; this is an assessed part of the funding application. There are also grant opportunities that specifically support researchers to engage in public involvement in their research (e.g. Medical Research Council public engagement seed funding). Furthermore, there are guidelines on inclusive autism research from autism-specific research funders (e.g. Autism Cooperative Research Centre in Australia; den Houting, 2021), as well as guidelines and requirements for national ethics bodies (e.g. the UK’s Health Research Authority/INVOLVE, 2016). These requirements are one critical way that research bodies can support the involvement and inclusion of community in autism research and provide a blueprint for research bodies in Aotearoa NZ.

### Strengths and limitations

This is the first study that has examined the distribution of autism research funding in Aotearoa NZ and, as such, provides a baseline for evaluating future funding trends. It is also one of the few studies to have included autistic people and the wider autism community in the study design, and to have actively sought feedback from these communities on the distribution of funding. In relation to seeking the views of the community, the project advisory groups were actively involved in the design of the online survey and focus groups, as well as the recruitment strategy and materials and question format, which improved the accessibility and relevance of these methods. The community partnership approach adopted in this study is also likely to have contributed to the diversity of autistic people in the survey respondents, including autistic people with co-occurring conditions such as intellectual disability. While this study goes some way to include the perspectives of those with intellectual disabilities in the discourse around autism research, future research should focus specifically on increasing the representation of autistic people with intellectual disability as well as those who may use non-traditional forms of communication, who may well have different views on the distribution of autism research funding.

This study was strengthened by the use of existing funding categories (IACC, 2017), which were used in the funding analyses from other countries, thus adding to our understanding of global autism research funding. However, this categorisation system also posed some limitations to the study. The IACC categories are not necessarily reflective of the unique cultural context or funding landscape of Aotearoa NZ (e.g. there was no category for Kaupapa Māori research) or community identified research priorities. The terminology of the IACC categories is biased towards a medical-deficit model of autism, which may have introduced variability in how members of the community interpreted these areas and how their own preferences aligned (or did not) with these different categories. While most grants appropriately aligned with these categorisations, there was some ambiguity about the most appropriate classification of some grants and/or forced categorisation (e.g. genetics research could span *Biology* and *Causes/Risk Factors*). Indeed, there is also likely to be some variation relating to the type of autism research even within categories. For example, the *Treatments and Interventions* category is inclusive of interventions that may seek to reduce ‘autism symptomology’, as well as interventions that seek to build the skills and strengths of autistic people. The small number of grants documented precludes a more nuanced analysis within the IACC categories. Future research should endeavour to co-design a specific coding system for the analysis of autism research funding, that is inclusive of community priorities and specifically monitors funding allocated in accordance with community priorities.

While the primary public funding bodies in Aotearoa NZ were captured, it is possible that a small number of grants awarded by private sector funders (e.g. philanthropic grant), small regional funders or those who would not ordinarily fund autism research were not captured in the current analysis. Likewise, this research focused specifically on contestable research grants. As such, commissioned research projects (i.e. by some government agencies) were not included in the search. It may be that a different distribution of funding may be observed from funding obtained through philanthropic donations and commissioned research. Furthermore, an examination of the distribution of unfunded projects, as well as funded projects, would permit a clearer understanding of whether the bias towards biological research extends to both funded and unfunded autism research projects. Such an examination may prove challenging, however, as it would require disclosure of potentially sensitive information from funders and/or researchers.

## Conclusion

While there is vast potential for advisory committees and research cooperatives in influencing future autism research, it is noteworthy that there is ongoing misalignment between funding distribution, the recommendations of existing advisory committees and the autistic and autism communities (den Houting & Pellicano, 2019; Harris et al., 2021). Further research into the basis of funding decisions, how advisory and community perspectives can be reflected in funding decisions and how to shift historical funding for autism research is needed.
